# Impact of Nutritional Supplements and Antioxidants in the Treatment of Breast Cancer: A Systematic Review

**DOI:** 10.3390/nu18091328

**Published:** 2026-04-23

**Authors:** Daniel Uribe-Ramírez, Kevin David Laguna-Maldonado, Melissa Vázquez-Carrada, Luis Fernando Cortés-Peña, María Magdalena Vilchis-Landeros, Héctor Vázquez-Meza, Deyamira Matuz-Mares

**Affiliations:** 1 Departamento de Bioquímica, Facultad de Medicina, Universidad Nacional Autónoma de México, Ciudad de México 04510, Mexico; danielur@bq.unam.mx (D.U.-R.); kevinlag@facmed.unam.mx (K.D.L.-M.); drlfcp.cortes@gmail.com (L.F.C.-P.); vilchisl@unam.mx (M.M.V.-L.); hvazquez@bq.unam.mx (H.V.-M.); 2 Cluster of Excellence on Plant Sciences, Institute for Microbiology, Heinrich Heine University Düsseldorf, 40225 Düsseldorf, Germany; m.vazquez-carrada@hhu.de

**Keywords:** breast cancer, dietary antioxidants, antioxidant vitamin supplement, oxidative stress

## Abstract

**Background/Objectives:** Dietary antioxidants are frequently utilized by breast cancer (BC) patients to mitigate treatment-related toxicities and enhance quality of life. However, their clinical efficacy remains highly controversial due to conflicting epidemiological and clinical data. This review aims to critically evaluate the molecular mechanisms, clinical outcomes, and translational challenges of antioxidant supplementation in BC management. **Methods:** A comprehensive evaluation of current literature—encompassing observational cohorts, randomized controlled trials, and mechanistic in vitro/in vivo models—was conducted. The analysis focused on the pharmacological interactions of diverse bioactive compounds (polyphenols, vitamins, carotenoids) with BC progression and standard antineoplastic regimens. **Results:** Current evidence demonstrates a paradoxical, double-edged role of antioxidants in oncology. While specific interventions (e.g., Coenzyme Q10, melatonin) effectively ameliorate treatment-induced toxicities without compromising therapeutic efficacy, the concurrent administration of antioxidants during cytotoxic chemotherapy can inadvertently neutralize essential reactive oxygen species (ROS), correlating with increased disease recurrence and mortality. Furthermore, clinical translation is severely hindered by the intrinsic hydrophobicity of natural compounds, the lack of whole-food matrix standardization, and dose-dependent hepatotoxicity. Emerging targeted delivery systems, such as lipid nanoformulations, show significant potential in overcoming these pharmacokinetic barriers. **Conclusions:** The therapeutic viability of antioxidant supplementation in BC is not universal; it is heavily dictated by intrinsic tumor biology, specific treatment modalities, and chronopharmacology. These findings underscore a critical biological imperative to transition from generalized dietary guidelines toward a rigorous paradigm of precision nutritional oncology, strictly avoiding concurrent antioxidant supplementation during active oxidative therapies.

## 1. Introduction

Currently, cancer represents one of the most significant public health challenges worldwide. It remains a leading cause of mortality, and its incidence is projected to rise due to population aging and the adoption of western lifestyles characterized by sedentary behavior, tobacco consumption, and poor dietary habits. Consequently, there has been growing interest in scientific research examining the relationship between dietary factors and cancer prevention [1,2]. In this context, an important approach is to analyze the role of antioxidants, particularly those derived from foods and dietary supplements, to establish effective strategies for the treatment and prevention of various types of cancer [1,3,4,5].

The World Health Organization (WHO) and the United Nations recognize breast cancer (BC) as the most prevalent malignancy worldwide. It accounts for approximately 2.3 million newly diagnosed cases annually, representing 11.6% of all cancer incidence [6,7,8]. Notably, BC is recognized as the second-leading cause of brain metastases, with the cerebellum alone accounting for 33% of such secondary occurrences [9]. Current literature suggests that the rising epidemiological burden of BC is heavily driven by modifiable risk factors. These include behavioral variables such as alcohol consumption, highly processed diets, physical inactivity, and obesity, as well as pharmacological factors like hormone replacement therapy and oral contraceptives. Therefore, in contrast to malignancies primarily driven by inherited genetic mutations, the increasing incidence of BC is strongly associated with these environmental and lifestyle factors [1,4,10,11,12,13].

Based on molecular and histological profiles, BC is broadly classified into three main subtypes: (i) hormone receptor-positive (HR+), which expresses estrogen (ER+) and/or progesterone (PR+) receptors; (ii) human epidermal growth factor receptor 2-positive (HER2+); and (iii) triple-negative breast cancer (TNBC), characterized by the lack of ER, PR, and HER2 expression (ER−/PR−/HER2−) [14,15]. Furthermore, TNBC is recognized as a highly heterogeneous disease that can be further subclassified into six distinct molecular subtypes: (a) basal-like 1 (BL1), (b) basal-like 2 (BL2), (c) immunomodulatory (IM), (d) mesenchymal (M), (e) mesenchymal stem-like (MSL), and (f) luminal androgen receptor (LAR) [15]. This profound heterogeneity significantly complicates clinical management and treatment protocols. Consequently, therapeutic strategies must be specifically tailored to the unique molecular and histological profile of the patient’s tumor [8,12].

The primary therapeutic modality for BC is surgical resection, frequently complemented by adjuvant chemotherapy and/or radiotherapy. For early-stage BC, clinical decision-making is largely guided by hormone receptor (ER/PR) and HER2 status, alongside tumor size and lymph node involvement. Conversely, in advanced or metastatic disease, the standard of care shifts to systemic approaches, encompassing chemotherapy, endocrine therapy, and targeted immunotherapy [4,12]. Chemotherapeutic treatments include various antimetabolites (agents that interact with DNA), hormones, and antitubulin agents [12,16,17]. The clinical efficacy of chemotherapy is severely compromised by the development of chemoresistance—an intrinsic or acquired ability of cancer cells to evade therapeutic agents—which frequently leads to clinical relapse. Additionally, the non-specific nature of these drugs results in off-target cytotoxicity to healthy tissues, ultimately impairing the patient’s overall quality of life [16,18].

Recently, researchers have investigated the use of nutritional supplements and antioxidants as adjunctive strategies in BC treatment. These interventions aim to optimize therapeutic efficacy, alleviate therapy-induced toxicities, and ultimately improve long-term overall survival [1,2,3,5,19,20,21]. Nevertheless, it is crucial to note that the current clinical application of these products relies predominantly on empirical evidence. For instance, preliminary data suggest that these compounds may mitigate treatment-induced adverse effects, including fatigue, nausea, mucositis, and anemia [18]. On the other hand, the importance of these biological sources is highlighted by the fact that 83% of chemotherapeutic agents approved by the Food and Drug Administration (FDA) are derived from natural products [10]. In this regard, medicinal mushrooms have emerged as a promising resource in integrative oncology, acting as biological response modifiers capable of enhancing immune function and potentially reducing chemo-induced myelosuppression [22]. Consequently, while existing literature supports the potential benefits of these supplements, robust, large-scale clinical trials are strictly required to definitively validate their efficacy and safety profiles in oncology patients.

Therefore, the main purpose of this systematic review is to evaluate the current clinical evidence regarding the impact of nutritional supplements on BC treatment outcomes. Additionally, this study aims to elucidate the underlying molecular mechanisms through which these compounds modulate breast tumor biology.

## 2. Method

Following the PRISMA protocol, an advanced search was conducted in PubMed and Cochrane Library using the following MeSH terms: (‘Breast Neoplasms’[Mesh] AND ‘Antioxidants’[Mesh]). The search was restricted to studies published between 2015 and 2026, identifying a total of 96 articles. In the initial review, 7 researchers identified 36 duplicate articles across both platforms and removed them. To ensure the quality of the evidence, the researchers performed a three-step screening process. First, the Newcastle-Ottawa Scale (NewCastle/Ottawa, NSW/ON, Australia/Canada) was applied; however, due to the nature of this instrument, it could not be applied to all identified articles. To complement the first tool, the AMSTAR 2 (Ottawa, ON, Canada) tool was additionally employed, as it also evaluates the methodological quality of review articles. Finally, for those results where neither scale was applicable, a qualitative review of methodological characteristics was conducted to ensure the inclusion of only high-quality studies. Subsequently, a content review was performed to verify alignment with the research question; through this process, 19 articles were excluded for not being directly related to the study objective.

**Figure 1:**
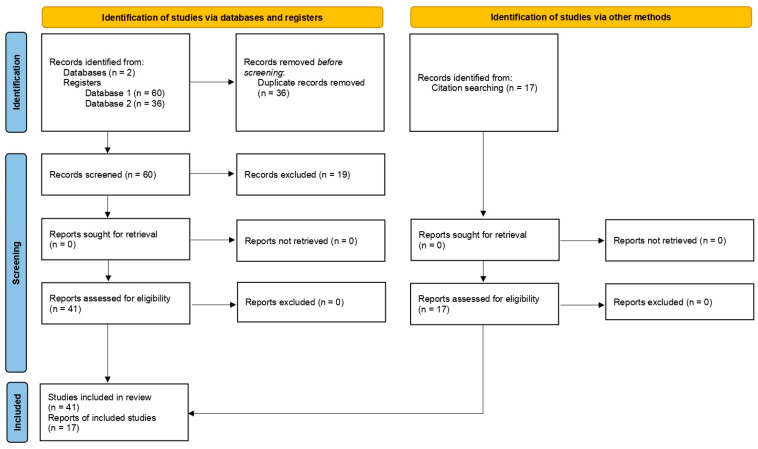
Flow diagram representing the study selection and categorization process for the search results of Breast Neoplasms and Antioxidants [23]. This work is licensed under CC BY 4.0. To view a copy of this license, visit https://creativecommons.org/licenses/by/4.0/.

The initial sample consisted of 41 articles that met the pre-established content review standards (Figure 1). Subsequently, a manual search (backward citation tracking) of the reference lists of these 41 articles was conducted to identify additional relevant studies not captured in the original database search. From this secondary review, 10 additional articles were included in the final selection.

To facilitate the analysis of this work, we chose to divide the findings from the 58 final articles into two major sections: the first addresses the molecular mechanisms of all antioxidant molecules recorded during the literature review; the second section summarizes the clinical findings reported for the antioxidant nutrients utilized across the various research articles.

## 3. Anticancer Mechanisms of Action of Nutritional Supplements and the Oxidative Stress Dilemma

BC progression and therapeutic response are intrinsically linked to the cellular redox state. Conventional oncological treatments primarily rely on cytotoxicity mediated by the excessive generation of reactive oxygen species (ROS) to eliminate neoplastic cells. In parallel, emerging therapeutic strategies increasingly incorporate nutritional supplements aimed at protecting healthy cells from the damage induced by antitumor agents, thereby reducing adverse effects in BC patients. However, this dual role of supplements creates a complex therapeutic scenario, as conventional treatments, particularly radiotherapy and certain chemotherapeutic agents, such as anthracyclines and alkylating agents, depend on the induction of lethal oxidative damage to tumor DNA and lipid membranes [19,24,25]. Whereas compounds such as Coenzyme Q10 (CoQ10), vitamins C and E, curcumin, quercetin, and sulforaphane possess an intrinsic capacity to neutralize these ROS [26,27]. In this way, nutritional supplements exert anticancer effects through a complex network of molecular mechanisms that interfere with all stages of carcinogenesis, from initiation to metastasis [25,27].

To evaluate the potential role of nutritional supplements in the oncological context, it is essential to understand the diverse biological mechanisms through which they may influence cancer cells. These mechanisms range from the direct neutralization of oxidative stress to the modulation of the tumor microenvironment and key cellular signaling pathways that regulate proliferation, programmed cell death, and tumor-associated inflammatory responses. The main mechanisms identified are described below (Table 1).

The primary mechanisms to consider involve antioxidant vitamin supplements (AVS), including vitamins C, D, and E, as well as carotenoids (e.g., lycopene) and polyphenols, which directly scavenge ROS to protect genomic DNA from oxidative mutations [25]. Furthermore, compounds such as quercetin activate nuclear factor erythroid 2-related factor 2 (Nrf2), a master transcriptional regulator that upregulates endogenous antioxidant enzymes, including superoxide dismutase (SOD) and catalase [1,8,19]. Conversely, in high doses, vitamin C can exhibit pro-oxidant activity by generating hydrogen peroxide. This accumulation overwhelms the limited antioxidant defenses of cancer cells, thereby inducing selective cytotoxicity without compromising healthy tissues [19,25].

Evasion of programmed cell death is a fundamental hallmark of cancer; however, targeted nutritional interventions can counteract this by reactivating apoptotic pathways [12,19,24]. For instance, supplements such as melatonin, resveratrol, and epigallocatechin gallate (EGCG) disrupt the balance between pro-apoptotic (Bax, Bak) and anti-apoptotic (Bcl-2) proteins. This modulation induces mitochondrial cytochrome c release, which subsequently activates the caspase cascade and promotes apoptotic cell death [12,30,31]. Furthermore, compounds such as vitamin E and melatonin have been reported to upregulate death receptor expression (e.g., Fas/FasL and TNF-related apoptosis-inducing ligand (TRAIL), thereby increasing tumor cell sensitivity to extrinsic apoptotic signaling pathways [4,25,45]. Similarly, selenium promotes apoptosis through the targeted generation of superoxide radicals followed by mitochondrial membrane potential loss [3].

Another critical mechanism exerted by nutritional supplements is their capacity to arrest uncontrolled cancer cell proliferation by targeting key cell cycle checkpoints [4,19]. For instance, phytochemicals such as genistein, kaempferol, and quercetin effectively downregulate the expression of cyclins (e.g., cyclins D1 and E) while simultaneously upregulating cyclin-dependent kinase (CDK) inhibitors, including p21, p27, and the tumor suppressor p53 [4,12]. Many supplements, such as resveratrol, quercetin, curcumin, and melatonin, block the PI3K/Akt/mTOR pathway, which is essential for growth and drug resistance [4,12,28,31].

In advanced disease stages, targeting angiogenesis and metastatic dissemination becomes a priority therapeutic objective [24,30]. Bioactive compounds such as melatonin, EGCG, and myricetin potently downregulate the expression of vascular endothelial growth factor (VEGF) and hypoxia-inducible factor 1-alpha (HIF-1α), thereby restricting tumor neovascularization and depriving the tumor of essential nutrients [12,19,24]. Additionally, polyphenols inhibit matrix metalloproteinases (specifically MMP-2 and MMP-9) enzymes responsible for extracellular matrix degradation, a prerequisite for tumor cell invasion and migration [28,30,34]. Furthermore, these supplements effectively suppress the epithelial–mesenchymal transition, a critical fundamental process driving the metastatic cascade [3,10,29].

Bioactive compounds such as sulforaphane and EGCG act as potent epigenetic modulators by inhibiting DNA methyltransferases (DNMTs) and histone deacetylases (HDACs), thereby reactivating transcriptionally silenced tumor suppressor genes [5,13,28,34]. Concurrently, polyphenols bolster the antitumor immune response by promoting the activation of cytotoxic T lymphocytes and Natural Killer (NK) cells. Furthermore, they attenuate the infiltration of immunosuppressive cells and facilitate the repolarization of tumor-associated macrophages (TAMs) toward an antitumoral phenotype [28].

In hormone receptor-positive malignancies, phytoestrogens such as the isoflavone genistein competitively bind to estrogen receptors, effectively dampening hormone-driven proliferative signaling [28,29]. Additionally, supplements like resveratrol and hesperidin disrupt tumor bioenergetics by inhibiting aromatase activity and impeding glucose uptake, thereby compromising the altered energy metabolism characteristic of cancer cells [12,41].

Ultimately, bacterial dysbiosis within both the gut and local breast tissue microbiomes represents a critical driver in breast cancer pathogenesis, orchestrated through a complex network of hormonal, metabolic, and immunological mechanisms [1]. Systemically, alterations in the estrobolome—the specific subset of gut microbiota responsible for estrogen metabolism and regulation—lead to elevated circulating estrogen levels, presenting a pronounced risk factor during menopause [46]. Locally, the depletion of commensal breast microbiota, particularly within the Lactobacillaceae family (e.g., *L. vini* and *L. paracasei*), fosters a pro-tumorigenic microenvironment [47]. This vulnerability is further exacerbated by the compromised biotransformation of dietary polyphenols (e.g., EGCG) into bioactive, antineoplastic metabolites—a highly specialized metabolic conversion strictly dependent on enzymatic activity from specific genera such as *Bifidobacterium* or *Clostridium* [37]. Consequently, dysbiosis significantly impairs local and systemic immune surveillance [1]. Reversing this microbial imbalance via targeted probiotic interventions, such as *Lactobacillus acidophilus* supplementation, holds substantial therapeutic promise for enhancing antitumor immune responses and promoting apoptosis in malignant cell [1,48]. This phenomenon is related to the gut microbiome’s ability to metabolize estrogens and the loss of immunological homeostasis; in this context, the use of probiotics (such as some *Lactobacillus* species and kefir) emerges as a promising therapeutic strategy due to their systemic immunomodulatory effects [1,21]. Mechanistically, these beneficial microbes confer active protection by stimulating effector populations, namely cytotoxic T lymphocytes and NK cells. Furthermore, they actively reshape the tumor microenvironment by driving the repolarization of tumor-associated macrophages from a pro-oncogenic M2 state toward a tumoricidal M1 phenotype [1,21,28].

## 4. Clinical Evidence for Dietary Interventions and Nutritional Supplementation in Breast Cancer Management

Nutritional management in breast cancer (BC) has definitively transcended the traditional palliative care paradigm, evolving into an advanced framework of nutritional pharmacodynamics. Current clinical evidence underscores that the therapeutic efficacy of bioactive compounds is not a static phenomenon; rather, it is critically governed by the precise intervention window or chronobiology relative to the administration of cytotoxic and biologic treatment protocols [1,27]. Under this premise, dietary supplementation must be conceptualized as an active modulator of the tumor microenvironment, capable of fundamentally altering the architecture of therapeutic response. While the elucidated biological mechanisms of various dietary supplements strongly suggest potential antitumor efficacy [12,24], the true clinical relevance of these bioactive compounds is ultimately contingent upon robust evidence derived from human studies, specifically prospective cohorts, randomized controlled trials, and meta-analyses. In recent years, numerous systematic reviews have investigated the association between nutritional supplementation and key clinical endpoints in BC patients, encompassing overall survival, disease-free survival, recurrence rates, and quality of life [4,26,27,32]. Current consensus from these comprehensive evaluations indicates a critical dichotomy: although specific micronutrients and phytochemicals may confer measurable benefits regarding life quality, mitigation of systemic inflammation, and reduction in therapy-induced toxicity, definitive evidence supporting their direct impact on long-term survival or tumor recurrence remains largely inconsistent. To address this paradigm, the following section provides a critical analysis of the clinical findings identified in our systematic review regarding the efficacy of nutritional supplements as an adjunct to standard BC treatment.

Regarding supplements exhibiting potent antioxidant properties, such as the AVS, clinical investigations have frequently evaluated multivitamin formulations. The pooled analysis indicated that AVS supplementation did not significantly compromise overall survival (HR = 0.92; 95% CI: 0.82–1.03). This is particularly critical when considering the redox antagonism observed during active cytotoxic therapy. In this context, landmark data derived from the SWOG S0221 cooperative group trial have established that concurrent antioxidant use—specifically Vitamins A, C, E, carotenoids, and Coenzyme Q10—during anthracycline- and taxane-based regimens significantly elevates the hazard of disease recurrence by 41% (HR = 1.41; 95% CI: 0.98–2.04) and overall mortality by 40% (HR = 1.40; 95% CI: 0.90–2.18) [49]. However, the authors highlighted an intriguing temporal heterogeneity within the data: whereas earlier studies suggested a potential survival benefit, more recent trials reported poorer prognostic outcomes among AVS users. A plausible explanation for this discrepancy involves unmeasured confounding variables in contemporary cohorts; specifically, patients opting for supplementation might exhibit suboptimal adherence to standard-of-care oncological therapies, ultimately leading to compromised survival outcomes [27].

On the other hand, the analysis of individual vitamins reveals a heterogeneous landscape, with effects varying considerably from one compound to another, both in terms of overall survival and disease recurrence. In the case of vitamin C, a meta-analysis of cohort studies that evaluated vitamin C intake after diagnosis found that its consumption was significantly associated with better overall survival (HR = 0.84, 95% CI: 0.76–0.93). However, most of the evidence comes from observational studies, so direct causality remains difficult to establish. On the other hand, the use of vitamin D has been widely investigated due to its role in regulating cell proliferation, differentiation, and apoptosis in breast tissues. Several observational studies have found that higher serum levels of vitamin D are associated with a significant reduction in mortality, estimated at approximately 42% in certain meta-analyses. However, randomized controlled clinical trials have not consistently demonstrated a protective effect of vitamin D on cancer prevention or prognosis, suggesting that elevated vitamin D levels may partially reflect better overall health status or healthier lifestyle factors rather than a direct causal relationship [1,32]. The integration of prognostic biomarkers allows for a rigorous personalization of nutritional interventions, where Vitamin D positions itself as a critical determinant; 25-hydroxyvitamin D levels ≥ 30 ng/mL after diagnosis are associated with a 42% reduction in specific mortality (HR = 0.58) [3]. Furthermore, antioxidant compounds may interfere with the ROS-mediated cytotoxic mechanisms by which chemotherapy eliminates tumor cells, potentially reducing treatment efficacy and even increasing toxicity (*p* < 0.01; *p* < 0.05). In contrast, in the post-diagnostic setting, interventions such as vitamin C supplementation have shown a markedly different effect, being consistently associated with reduced overall toxicity and improved survival outcomes (*p* < 0.01) [49].

The evidence regarding vitamin E is less conclusive. The available meta-analyses have not identified a significant association between vitamin E supplementation and overall survival in BC patients (HR = 0.94, 95% CI: 0.79–1.13). Nevertheless, some studies and reviews have suggested that certain forms of vitamin E, particularly tocotrienols and specific tocopherols, could exert relevant biological effects on tumor progression, including inhibition of cell proliferation, modulation of signaling pathways, and antiinflammatory effects. Despite these promising findings, the clinical evidence is not yet sufficient to establish clear therapeutic recommendations [10,27].

Overall, the available evidence suggests that the impact of antioxidant vitamins on BC survival is highly heterogeneous and appears to depend on the specific compound, the clinical context, and the timing of supplementation. While certain micronutrients, such as vitamin C, have shown favorable associations in observational cohort studies, others, including vitamin E, have demonstrated neutral effects on survival outcomes. Furthermore, the clinical utility of vitamin D remains highly debated due to persistent discrepancies between observational data and randomized controlled trials [1,27,32,49]. Therefore, well-designed prospective randomized controlled trials are needed to definitively clarify the prognostic role of vitamin supplementation in BC.

Transitioning to phytochemicals, the clinical impact of carotenoids on BC is multifaceted and appears highly contingent upon the intrinsic tumor subtype and HR status. These findings underscore the critical importance of considering tumor biological heterogeneity when evaluating the efficacy of micronutrients. Epidemiological evidence indicates that carotenoid lipophilic antioxidants abundant in fruits and vegetables may influence mammary carcinogenesis through the attenuation of oxidative stress, the modulation of intracellular signaling pathways, and the induction of apoptosis [24,28].

A comprehensive re-evaluation of a pooled analysis comprising 18 prospective cohort studies assessing circulating carotenoid concentrations revealed subtype-specific heterogeneity. Notably, all five primary carotenoids evaluated—α-carotene, β-carotene, lycopene, lutein, and zeaxanthin—consistently demonstrated protective associations against ER− breast malignancies. Specifically, elevated serum lycopene levels correlated with a significant reduction in overall BC risk (RR = 0.86). This risk attenuation is likely mediated by lycopene’s potent antioxidant capacity, coupled with its ability to downregulate proinflammatory and proliferative pathways [32,50]. Moving beyond the scope of direct cytotoxicity, emerging evidence underscores the critical role of the gut-breast axis and the modulation of the estrobolome via high-density dietary fiber interventions (≥25 g/day). This nutritional strategy is significantly associated with a reduction in circulating estrogen fractions, a fundamental determinant in the prognostic trajectory of estrogen receptor-positive (Luminal A and B) phenotypes [17,40,51]. Upon transitioning to the post-adjuvant survivorship phase, the role of micronutrients is redefined toward the restoration of systemic homeostasis and the mitigation of residual toxicity. The LACE cohort (n = 2264) demonstrated that high carotenoid intake, initiated strictly after the cessation of primary therapy, reduces the risk of recurrence by 33% (HR = 0.67; 95% CI: 0.50–0.91) [52], while the MARIE study suggests that this post-diagnosis supplementation improves overall survival by mitigating non-oncological mortality (HR = 0.73). This benefit is predominantly attributed to the protection of healthy parenchyma against the persistent adverse sequelae of conventional therapeutics.

However, some results indicate a possible differential effect depending on the tumor’s hormonal profile. β-carotene has been associated with a slight increase in the risk of hormone receptor-positive BC, including ER+ and ER+/PR+ tumors, with approximate effect sizes of 1037 and 1034, respectively. This finding suggests that the biological effects may be influenced by the hormonal context of the tumor. A proposed hypothesis is that, in ER+ tumors, β-carotene could interact with estrogen-dependent signaling pathways or influence cellular redox balance, favoring a tumor microenvironment that promotes cell proliferation. In contrast, in ER-tumors, where hormonal pathways do not play a dominant role, the antioxidant and antiproliferative properties of carotenoids could predominate, contributing to a protective effect [24,28].

These findings show that the effects of carotenoids are not uniform and can vary depending on the molecular subtype of BC, circulating concentrations of the compounds, and interactions with specific metabolic and hormonal pathways. Consequently, the available evidence supports the need for a more personalized and subtype-stratified approach when evaluating the role of carotenoids in BC prevention and prognosis.

Beyond vitamins and carotenoids, other dietary compounds and bioactive supplements have also been associated with the risk of recurrence, tumor progression, and BC development. These compounds include polyphenols, probiotics, specific micronutrients, and plant-derived metabolites that can act through antioxidant, antiinflammatory, immunomodulatory, or epigenetic mechanisms.

For example, green tea is one of the most studied dietary sources of polyphenols, particularly catechins like EGCG. Epidemiological studies and systematic reviews have suggested that its consumption can be associated with a reduced risk of BC recurrence, with a pooled relative risk estimate of 0.73. Proposed mechanisms include the inhibition of cell proliferation, induction of apoptosis, and modulation of signaling pathways related to tumor growth and angiogenesis [28,34].

Furthermore, the gut microbiome exerts a profound regulatory influence on the therapeutic efficacy of anti-PD-L1 immune checkpoint inhibitors, particularly in the context of TNBC. In this aggressive subtype, polyphenol-driven enhancement of microbial diversity optimizes the recruitment of tumor-infiltrating lymphocytes, thereby significantly improving pathological complete response rates [16,36,53]. Furthermore, marine-derived omega-3 polyunsaturated fatty acids (PUFAs) predominantly sourced from fatty fish have been extensively linked to potent anti-inflammatory and signal-modulating properties. Current observational data indicate that elevated dietary intake of these PUFAs is associated with a clinically significant 30% reduction in overall BC risk (RR ≈ 0.70). Omega-3 fatty acids show potential in the sensitization of HER2+ tumors and in the reduction in systemic inflammation measured by high-sensitivity CRP. Mechanistically, it is hypothesized that these long-chain lipids actively alter cellular membrane architecture (e.g., lipid raft microdomains), modulate intracellular signal transduction, and shift eicosanoid metabolism toward pro-resolving inflammatory mediators, collectively fostering a robust antitumoral environment [32,42].

In parallel with lipid-based interventions, experimental evidence has highlighted the therapeutic potential of fermented matrices, such as kefir extracts (*p* < 0.05). These microbial consortia have been shown to effectively inhibit the proliferation of estrogen-dependent malignant cells, likely through the synergistic action of fermentation-derived metabolites, bioactive exopolysaccharides, and milk-derived functional peptides. Moreover, comprehensive recent reviews underscore their capacity to reshape the gut microbiome ecosystem, prime the host’s systemic immune response, and dampen chronic inflammation, crucial indirect pathways that confer protection against tumor initiation and subsequent progression [1,21].

Moreover, a wide array of plant-derived phenolic compounds remains under intense investigation for their chemopreventive and therapeutic utility in breast oncology. Prominent molecules within this class—including the stilbene resveratrol and flavanols such as quercetin exhibit profound pleiotropic antineoplastic effects in experimental models, characterized by the robust induction of apoptosis, suppression of cellular proliferation, and attenuation of inflammatory cascades. Importantly, current systematic reviews posit that these phytochemicals function as pivotal epigenetic and immunological regulators, directly modulating the transcriptional landscape governing mammary carcinogenesis [4,28,30,31].

Furthermore, selenium has shown potential in modulating oxidative stress and regulating antioxidant enzymes involved in cellular defense, particularly in aggressive subtypes like TNBC. Finally, the maintenance of optimal selenium levels acts as a modulator in patients with TNBC, where its deficiency is closely linked to phenotypes of greater biological aggressiveness and metastatic progression [3]. For its part, CoQ10 has been studied for its role in mitochondrial function and protection against oxidative damage, in addition to its possible contribution to reducing adverse effects associated with oncological treatment [3,26]. Notably, the administration of CoQ10 alongside vitamin E derivatives has proven highly efficacious in mitigating anthracycline-induced cardiotoxicity. Moreover, combinatorial interventions involving CoQ10, specific carotenoids, and PUFAs have been reported to significantly alleviate cancer-related chronic fatigue (*p* < 0.05). Likewise, the polyphenol resveratrol has gained attention for its ability to attenuate the mental decline that occurs after chemotherapy, commonly called chemobrain (*p* < 0.05) [20,26,39]. Within this supportive care framework, targeted supplementation governed by strict dosing protocols has emerged as an efficacious strategy for managing dose-limiting toxicities. Specifically, Coenzyme Q10 (100–200 mg/day) prevents the decline in left ventricular ejection fraction (LVEF) induced by anthracycline and anti-HER2 regimens; silymarin (140 mg, three times a day) demonstrates a validated hepatoprotective effect by significantly lowering serum transaminases during doxorubicin administration; and resveratrol (500–1000 mg/day) mitigates the neuroinflammation underlying chemotherapy-induced cognitive impairment (chemobrain), thereby optimizing executive function following cytotoxic exposure.

In parallel, robust experimental models and clinical reviews highlight melatonin as a potent adjuvant agent endowed with pleiotropic oncostatic properties. These include the resynchronization of circadian rhythms, the downregulation of estrogen-dependent signaling cascades, and the potentiation of host immunosurveillance. Crucially, exogenous melatonin supplementation is postulated to significantly enhance patient tolerance to systemic antineoplastic therapies while actively buffering their associated adverse profiles [12,41].

Beyond their theoretical impact on long-term survival and recurrence, the clinical utility of bioactive supplements is increasingly defined by their capacity to manage treatment-related adverse events encompassing debilitating fatigue, systemic inflammation, cardiotoxicity, and therapy-induced metabolic dysregulation. While contemporary reviews suggest that targeted nutritional interventions can profoundly improve overall quality of life and therapeutic adherence, definitive confirmation of their direct efficacy on hard oncological endpoints still necessitates rigorous validation via large-scale randomized controlled trials [32,42].

Ultimately, when evaluating nutrient delivery systems, current evidence strongly supports the superiority of whole-food matrices over isolated pharmacological supplementation. Comprehensive dietary paradigms, notably the Mediterranean Diet and other polyphenol-dense regimens, consistently outperform singular supplements in elevating systemic total antioxidant capacity (*p* < 0.002) [5,38]. This phenomenon underscores the concept of “food synergy,” suggesting that obtaining diverse dietary antioxidants through a comprehensively balanced diet provides a safer, more highly effective strategy for buffering oncological toxicities.

As delineated in Table 2, specific dietary antioxidants must transcend the label of mere “supplements” and be repositioned as strategic therapeutic adjuvants. Current data consistently affirm that, when implemented under stringent clinical surveillance and administered within the optimal therapeutic window, these bioactive interventions effectively mitigate end-organ toxicity and maximize the tolerability of standard oncological regimens.

## 5. Discussion and Conclusions

Despite promising evidence for certain compounds, translating these findings into standard clinical practice faces significant obstacles, mainly due to the duality of their effects. Factors such as the low bioavailability of bioactive compounds and the marked heterogeneity of both the disease and the patients complicate the formulation of universal recommendations.

Bioavailability refers to the proportion of an administered compound that reaches systemic circulation and is available to exert a biological effect. This is one of the biggest obstacles for many promising phytochemicals. Curcumin is a paradigmatic example: despite demonstrating potent anticancer effects in laboratory studies (in vitro), its clinical utility is severely limited by its very low bioavailability when administered orally. Likewise, compounds like EGCG (from green tea) face low stability, limited intestinal absorption, and rapid metabolism, which reduces their effective concentration at the tumor site [34,37].

To overcome the limitations associated with poor bioavailability, current research is focused on developing innovative strategies to enhance the absorption, stability, and targeted delivery of these compounds, including combination therapies and nanoformulation-based delivery systems. The co-administration of certain compounds can drastically improve the absorption of others. For example, the bioavailability of curcumin improves significantly when combined with piperine, the active compound in black pepper. However, the use of vitamin B12 and iron during chemotherapy is significantly associated with worse disease-free survival and overall survival [49].

On the other hand, pharmaceutical technology offers promising solutions. The use of nanomaterials allows for the controlled administration of drugs with adjustable size, surface charge, and morphology [59]. Nanoparticles improve the bioavailability, stability, and solubility of antioxidants, as well as their localization in tumor cells [60]. Furthermore, they act as efficient delivery vehicles for antioxidants, improving their activity and overall efficacy [61].

Furthermore, the use of ligands or antibodies on these particles improves efficacy and reduces unwanted effects [62]. For example, curcumin nanoformulations, which encapsulate the compound in nanometric particles, have demonstrated a significant reduction in tumor volume in animal models, overcoming the limitations of commercial curcumin [1].

Conversely, quercetin, a polyphenol characterized by a broad spectrum of biological activities including antioxidant, antiviral, antidiabetic, antifungal, cardioprotective, and anticancer effects [30], exhibits limited clinical applicability due to its poor solubility and low bioavailability. Consequently, various strategies, including structural modification and nano- or microemulsion-based delivery systems, have been proposed to enhance its bioavailability and improve its therapeutic potential [4,29].

Another important challenge is related to the intrinsic variability of food-derived compounds. For instance, kefir has demonstrated considerable potential to inhibit tumor cell proliferation and induce apoptosis; however, its antioxidant capacity varies significantly depending on the composition of the microbial consortium, the type of milk used, and fermentation time. Such variability complicates the establishment of standardized therapeutic doses [21]. Furthermore, toxicity represents an additional concern, as natural compounds are not necessarily harmless and high doses may lead to adverse effects. For example, concentrated green tea extracts have been associated with elevated liver enzyme levels in some postmenopausal women [36].

It is necessary to consider that the efficacy of a supplement is not universal; it depends on a complex interaction of multiple factors, which demands a more personalized approach [32]. The clinical impact of micronutrient supplementation is strongly influenced by intrinsic tumor biology. The behavior of carotenoids serves as a quintessential paradigm: while they confer protective effects against ER− malignancies, they are potentially deleterious in the context of ER+ tumors [63]. Furthermore, the timing of supplementation is important since there is a critical difference between the use of antioxidants during chemotherapy, which is controversial and potentially harmful [49], and post-treatment supplementation, which is generally unassociated with adverse survival outcomes [27]. Finally, discrepancies in study design severely confound the current literature. While prospective observational cohorts frequently report favorable associations, they are fundamentally incapable of establishing causality. Conversely, randomized controlled trials often yield equivocal or null results—as prominently observed in vitamin D trials for cancer chemoprevention—highlighting the profound methodological complexities of proving causal relationships in nutritional epidemiology [43].

Moreover, converging clinical and observational evidence highlights that the timing or chronobiology of supplementation is a critical determinant of clinical outcomes. The administration of antioxidants concurrent with chemotherapy or radiotherapy has brought out profound clinical concern. The underlying rationale is that exogenous antioxidants may inadvertently neutralize the oxidative stress deliberately induced by cytotoxic treatments, which constitutes the primary mechanism of action for tumor cell eradication in these modalities. Consequently, landmark investigations involving BC cohorts undergoing active chemotherapy have demonstrated that concurrent antioxidant supplementation is significantly associated with an elevated hazard of disease recurrence and overall mortality, in comparison with patients who did not receive supplementation during active treatment [49].

**Figure 2:**
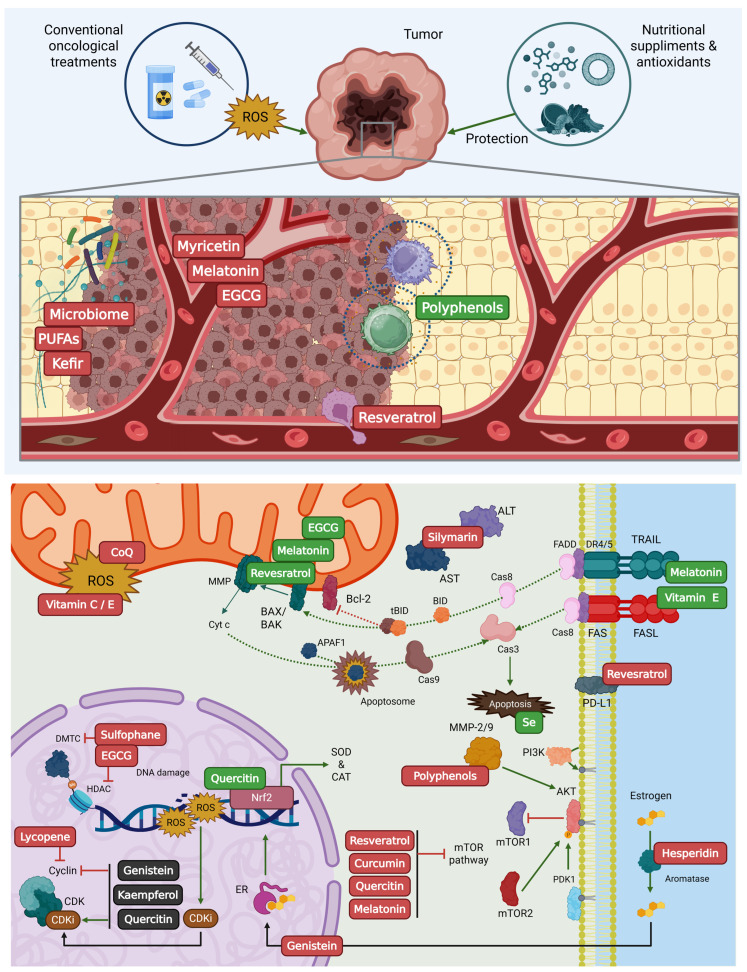
Mechanism of action of nutritional supplements in the physiology of cancer cells. **Intercellular effects (upper panel):** Polyphenols trigger the activation of cytotoxic T lymphocytes and Natural Killer (NK) cells, as denoted by the circle encompassing these cell populations. Moreover, the microbiome, PUFAs, and kefir confer active protection by stimulating the immune response and reshaping the tumor microenvironment. In addition, melatonin, epigallocatechin gallate (EGCG), and myricetin potently restrict tumor neovascularization, while resveratrol inhibits the proliferation and metastasis of cancer cells. **Intracellular effects (lower panel):** Coenzyme Q and vitamins C and E neutralize reactive oxygen species (ROS) due to their intrinsic antioxidant capacity. Melatonin, resveratrol, and EGCG regulate the balance between pro-apoptotic (Bax, Bak, Cytc) and anti-apoptotic (Bcl-2) proteins. Silymarin reduces hepatic transaminases (AST, ALT). Furthermore, vitamin E and melatonin upregulate the expression of Fas/FasL and the TNF-related apoptosis-inducing ligand (TRAIL) by activating executioner caspases, such as Cas3. Selenium promotes apoptosis through the targeted generation of superoxide radicals. Polyphenols inhibit matrix metalloproteinases, and resveratrol downregulates Programmed Death Ligand 1 (PD-L1) expression. Resveratrol, quercetin, curcumin, and melatonin block the PI3K/Akt/mTOR pathway. Hesperidin disrupts tumor bioenergetics by inhibiting aromatase activity. Genistein competitively binds to estrogen receptors, dampening hormone-driven proliferative signaling. Sulforaphane and EGCG act as histone deacetylase (HDAC) and DNA methyltransferase (DNMT) inhibitors. Quercetin activates nuclear factor erythroid 2-related factor 2 (Nrf2), a master transcriptional regulator that upregulates endogenous antioxidant enzymes, including superoxide dismutase (SOD) and catalase. Genistein, kaempferol, and quercetin downregulate the expression of cyclins while upregulating cyclin-dependent kinase (CDK) inhibitors. Lycopene also acts as a cyclin inhibitor. Further information and abbreviations can be found in the text.

Considering these complexities, rigorous future investigations are mandated to surmount current translational hurdles and fully harness the adjuvant potential of dietary antioxidants in breast oncology. The observed variability in clinical outcomes is not merely a methodological artifact but a fundamental biological imperative that necessitates a definitive shift from generalized dietary guidelines toward a rigorous paradigm of precision nutritional oncology. Currently, the landscape of evidence is frequently compromised by pervasive methodological limitations, including underpowered sample sizes, highly variable dosing regimens and supplementation durations, and a failure to adequately control for critical confounding variables such as baseline dietary patterns, lifestyle factors, and concurrent allopathic treatments [1,32]. As a result of this work, we made a graphic resume in the Figure 2.

## Figures and Tables

**Table 1:** Antioxidant compounds and their dietary sources and activities.

Bioactive Compound	Dose	Administration Time	Antineoplastic Activities	Primary Dietary Sources	References
Quercetin	2000 mg/m^2^ (IV) or 30–34 mg/kg dose (in preclinical models).	Variable; evaluated in 2-week protocols or chemotherapy cycles.	Antiestrogenic, tyrosine kinase inhibitor, and inhibitor of angiogenesis and metastasis. Activate Nrf2 and block the PI3K/Akt/mTOR pathway.	Apples, onions, berries, leafy greens, citrus fruits, potatoes, tomatoes, lettuce, celery, eggplant, tea, and wine.	[1,4,10,25,28,29,30]
Resveratrol	Variable concentrations	Primarily evaluated in preclinical models	Induces apoptosis, inhibits proliferation and metastasis, reduces PD-L1 expression, blocks the PI3K/Akt/mTOR pathway, and activates cytotoxic T lymphocytes and NK cells.	Grapes, red wine, berries (blueberries, blackberries), peanuts, cocoa, pistachios, and itadori (*Poligonum cuspidatum*).	[5,10,28,29,31]
Curcumin	200 mg/day	For ≥ 3 months.	Antiproliferative, antitumoral, apoptosis inducer, antiangiogenic, blocks the PI3K/Akt/mTOR pathway and activates cytotoxic T lymphocytes and NK cells.	Turmeric (*Curcuma longa*).	[1,10,24,25,28,29,32]
EGCG	1 g (twice a day) or 843 mg daily.	12 to 18 months (oral route)	Antitumoral, antiangiogenic, DNA methylation and Telomerase Reverse Transcriptase (TERT) inhibitor, apoptosis inducer, chemopreventive, and radioprotective. inhibiting DNA methyltransferases (DNMTs) and histone deacetylases (HDACs),	Green tea (*Camellia sinensis*) and white tea.	[1,10,28,29,32,33,34,35,36,37]
Lycopene	58 mg of β-carotene (in mixture) or 6 mg/day (nutritional)	Evaluated in 24-month follow-ups or short 7-day periods	Antiproliferative, apoptosis inducer, cyclin D1 suppressor, inhibitor of metastasis and angiogenesis, and protective against splenic damage.	Tomatoes and watermelon.	[1,10,24,25,26,38]
Vitamin C	120 mg/day (nutritional) or up to 2800 mg/day (in mixtures).	Administered continuously post-diagnosis or for 24 months.	Selective cytotoxicity against cancer cells, mitigates chemotherapy toxicity, neutralizes free radicals, and improves overall survival in BC.	Citrus fruits, kiwi, strawberries, broccoli, bell peppers, Brussels sprouts, and kefir.	[1,19,21,24,25,26,27,32,38,39]
Genistein	138 mg (soy isolate)	5 days (in post-treatment pilot study).	Estrogen receptor modulator, phytoestrogen, antioxidant, tyrosine kinase inhibitor, apoptosis inducer, and inhibitor of cell growth and angiogenesis, affecting key signaling pathways such as PI3K/Akt and MAPK. This includes suppressing protein levels like MEK5 (mitogen-activated protein kinase 5) and ERK5 (extracellular signal-regulated kinase 5).	Soybeans, tofu, and red clover.	[5,28,29,30]
Kaempferol	Variable concentrations (in vitro).	Primarily evaluated in preclinical models	Antineoplastic (breast, prostate, lung) and antiangiogenic. Decreases the expression of cyclins and increases the expression of cyclin-dependent kinase (CDK) inhibitors.	Beans, broccoli, cabbage, grapes, apples, citrus fruits, strawberries, and tomatoes.	[4,29]
Hesperidin/Hesperetin	The exact human clinical dose is not specified in the sources.	Preclinical/Perimenopause.	Aromatase inhibitor and hypocholesterolemic	Citrus fruits (lemon, lime, orange, grapefruit) and tomatoes.	[4,29]
Sulforaphane	Equivalent to 234 g of broccoli sprouts daily	72 h (in vitro) or long-term administration.	HDACs inhibitor decreases the expression of DNA methyltransferases (DNMT1 and DNMT3A), antineoplastic, and mammary tumorigenesis preventive.	Broccoli sprouts, cruciferous vegetables (cabbage, kale, Brussels sprouts).	[1,10,13]
β-Carotene (Provitamin A)	58 mg (in antioxidant mixture).	Evaluated in 24-month follow-up or short 7-day periods.	Cyclin D1 suppression and cell cycle arrest. The mixture with β-carotene was associated with tumor remission.	Carrots, yellow/orange vegetables, and kefir.	[1,5,19,21,24,26,38,39,40]
Melatonin	20 mg/day (oral route).	Generally, 1 year or starting 7 days before hormone therapies.	Antitumoral and oncostatic, regulates the balance between Bax, Bak and Bcl-2 proteins. Upregulates death receptor expression.	Endogenously produced (pineal gland), available as a pharmacological supplement.	[12,32,41]
Coenzyme Q10	100–390 mg/day; low doses of 30 mg for fatigue.	From 3 weeks (for fatigue) up to 1–2 years as an adjunct.	DNA protector mitigates chemotherapy-induced cardiotoxicity and reduces fatigue. Reduce marcadores tumorales (CEA, CA 15-3).	Fish, meat, vegetables, fruits, nuts, and oils (also endogenous production).	[10,26,42]
Myricetin	Concentraciones variables (in vitro).	Durante el periodo de tratamiento con quimioterapia.	Antineoplastic, reduces the activity of matrix metalloproteinases MMP-2 and MMP-9, limiting the invasive and metastatic capacity of the tumour; it also blocks the PI3K/Akt/mTOR pathway.	Myricaceae, Anacardiaceae plants, vegetables, fruits, and teas.	[4]
Vitamin E	600 mg (3 times a day) or 30 mg/day (nutritional).	During the treatment period or up to 30 weeks (in animal models).	Reduce tumor volume, inhibits cell proliferation (colon and breast), increases the levels of pro-apoptotic proteins such as Bax, and activates caspases 3, 8, and 9.	Germs of corn, wheat, canola, flaxseed, sesame, soybeans and them oils.	[10,38,42]
Vitamin D	0.5 µg/day or 50,000 IU (weekly) or a single dose of 200,000 IU.	Throughout the entire chemotherapy cycle or for 2–3 months.	Cardioprotective against doxorubicin toxicity,	Endogenous synthesis (UV exposure), fortified dairy, fatty fish, and egg yolks.	[10,32,42,43]
Omega-3 PUFA	4 g/day of fish oil; 3.5 g in nutritional blends.	3 months (bone health) or continuous post-diagnosis use.	Stimulates the immune response and the tumor microenvironment. Increases the levels of caspase-3 and p53 and reduces the expression of the anti-apoptotic protein Bcl-2	Oily fish, fish oil, cod liver oil, flax seeds, nuts and other seeds	[1,26,32]
Selenium	100 µg (nutritional) or 385 µg (in therapeutic mixtures).	Post-diagnosis (24 months) or prevention (7–8 years).	Acts as a pro-oxidant, inducing death by apoptosis, necrosis, or autophagy, induces cell cycle arrest (G2/M phase), and inhibits angiogenesis and EMT.	Fish, shellfish, seeds and nuts.	[19,26,27,38]
Silymarin	420 mg/day (divided into 3 doses of 140 mg).	63 consecutive days since the start of chemotherapy.	Promotes apoptosis, reduces hepatic transaminases (AST, ALT), suppresses angiogenesis and blocks EMT by downregulating MMPs.	*Silybum marianum*.	[29,32,44]

**Table 2:** Clinical Interventions for Symptom Management and Mitigation of Oncology Treatment-Related Toxicities.

Clinical Focus/Target Symptom	Intervention/Compound	Potential Benefit (Evidence)	Precautions/Potential Adverse Effects	Timing of Administration	Statistical Significance	References
Fatigue and Quality of Life	Coenzyme Q10	Significant reduction in treatment-related fatigue; improvement in systemic antioxidant markers.	Generally safe, but interactions with specific chemotherapeutic agents must be monitored if utilized at very high doses during active treatment.	Pre- and concurrent chemotherapy.	*p* < 0.05	[26,39]
Sleep Disorders and Depression	Melatonin	Improves sleep quality and mitigates depressive and anxiety symptoms. Acts as an antioxidant and may synergize with chemotherapy by reducing neurotoxicity.	No frequent severe adverse effects reported; considered a highly protective agent with dual antineoplastic potential.	Concurrent with treatment.	*p* ≤ 0.05; *p* ≤ 0.01	[12,41]
Cognitive Impairment (“Chemobrain”)	Resveratrol	Neuroprotective potential against chemotherapy-induced neuroinflammation; enhances neuronal membrane integrity and cognitive function. Associated with memory and executive function improvements in survivors.	High doses may exhibit anticoagulant effects. Controversial use during concurrent oxidative chemotherapy.	Concurrent and post-treatment.	*p* < 0.05	[54,55]
Cardiotoxicity	Coenzyme Q10 and Omega-3 PUFAs	Nutritional modulation to mitigate cardiac damage induced by anthracyclines (e.g., doxorubicin) and anti-HER2 therapies (e.g., trastuzumab).	Low oral bioavailability limits clinical efficacy in certain contexts; advanced delivery systems (e.g., nanoparticles) are required to maximize the therapeutic effect.	Pre- and concurrent treatment.	*p* < 0.05	[42,56]
Gastrointestinal Health	Probiotics (kefir)	Enhances gastrointestinal health, reduces systemic inflammation, and improves quality of life in colorectal and gastric cancer patients.	None specified.	Pre- and concurrent treatment.	*p* < 0.05	[21,57]
General Antioxidant Use	Vitamins A, C, E, D, Carotenoids, Vitamin B12	Post-treatment: Vitamin C is associated with improved overall survival. Helps replenish micronutrient deficiencies. Vitamin D3 significantly increases serum Total antioxidant capacity.	Concurrent Chemotherapy: Antioxidant use (particularly vitamin B12, iron, and Omega-3) is associated with an increased risk of recurrence and mortality (e.g., SWOG S0221 trial) by neutralizing the oxidative mechanisms of cytotoxic agents (e.g., cyclophosphamide).	Pre-, concurrent, and post-treatment.	*p* < 0.05; *p* < 0.01; *p* = 0.004	[27,43,49]
Oxidative Stress and Systemic Nutrition	Mediterranean Diet	Enhances serum total antioxidant capacity and body composition; reduces the risk of comorbidities (e.g., obesity, metabolic syndrome) that negatively impact oncological prognosis.	None reported; widely considered the safest and most universally recommended lifestyle intervention.	Pre-, concurrent, and post-treatment.	*p* < 0.002	[5,39]
Hepatotoxicity	Silibinin/Silymarin, Green Tea Extract	Silibinin: Prevents hepatic steatosis and reduces hepatic transaminases (AST, ALT) post-doxorubicin. Nanoparticle delivery reduces systemic toxicity by targeting the tumor and protecting healthy tissue.	Green Tea Extract: Significant elevation of hepatic transaminases and increased risk of hepatotoxicity at highly concentrated doses.	Concurrent treatment.	*p* < 0.05	[36,44]
Nephrotoxicity	Quercetin	Demonstrated significant attenuation of cisplatin-induced renal toxicity (nephrotoxicity).	Low oral bioavailability. Extremely high intravenous doses may elicit off-target effects, though dietary intake remains safe.	Concurrent treatment.	*p* < 0.05	[30,58]
General Toxicity and Efficacy	Selenium	Utilized as an adjuvant to mitigate general chemotherapy-induced adverse effects while potentiating tumor cytotoxicity (particularly in TNBC).	Narrow therapeutic index; excessive intake induces toxicity (selenosis). Precise dosing is imperative to reduce adverse effects without eliciting intrinsic toxicity.	Concurrent treatment (Adjuvant therapy).	*p* < 0.05	[3,51]

## Data Availability

No new data were created or analyzed in this study. Data sharing is not applicable to this article.

## Abbreviations

The following abbreviations are used in this manuscript:

BC	Breast Cancer
WHO	World Health Organization
HR	Hormone Receptor
ER	Estrogen Receptor
PR	Progesterone Receptor
HER2	Human epidermal growth factor receptor 2
TNBC	Triple-negative breast cancer
BL-1	Basal-like 1
BL-2	Basal-like 2
IM	Immunomodulatory
M	Mesenchymal
MSL	Mesenchymal stem cell-like
LAR	Luminal androgen receptor
FDA	Food and Drug Administration
AVS	Antioxidant vitamin supplements
ROS	Reactive Oxygen Species
CoQ10	Coenzyme Q10
PD-L1	Programmed Death Ligand 1
EGCG	Epigallocatechin-3-gallate
TERT	Telomerase Reverse Transcriptase
HDAC	Histone deacetylases
Nrf2	Nuclear factor erythroid 2-related factor 2
SOD	Superoxide Dismutase
TRAIL	TNF-related apoptosis-inducing ligand
CDK	Cyclin-Dependent kinase
VEGF	Vascular endothelial growth factor
HIF-1α	hypoxia-inducible factor 1-alpha
MMP	Matrix metalloproteinases
DNMTs	DNA methyltransferases
NK	Natural Killer
TAMs	Tumor-associated macrophages
DNMTs	DNA methyltransferase
PUFAs	Polyunsaturated fatty acids
AST	Aspartate Aminotransferase
ALT	Alanine Aminotransferase

## References

[1] Mokbel K., Mokbel K. (2024). Harnessing Micronutrient Power: Vitamins, Antioxidants and Probiotics in Breast Cancer Prevention. Anticancer Res..

[2] Shah S., Laouali N., Mahamat-Saleh Y., Biessy C., Nicolas G., Rinaldi S., Zamora-Ros R., Papadimitriou N., Morales-Berstein F., Dahm C.C. (2025). Plant-Based Dietary Patterns and Breast Cancer Risk in the European Prospective Investigation into Cancer and Nutrition (EPIC) Study. Eur. J. Epidemiol..

[3] Sidira D., Siafaka A., Chrysikos D., Papadopoulos G., Stratopoulos E., Filippou D. (2024). Selenium and Triple Negative Breast Cancer. Acta Medica Acad..

[4] Wendlocha D., Krzykawski K., Mielczarek-Palacz A., Kubina R. (2023). Selected Flavonols in Breast and Gynecological Cancer: A Systematic Review. Nutrients.

[5] Braakhuis A., Campion P., Bishop K. (2016). Reducing Breast Cancer Recurrence: The Role of Dietary Polyphenolics. Nutrients.

[6] Bray F., Laversanne M., Sung H., Ferlay J., Siegel R.L., Soerjomataram I., Jemal A. (2024). Global Cancer Statistics 2022: GLOBOCAN Estimates of Incidence and Mortality Worldwide for 36 Cancers in 185 Countries. CA Cancer J. Clin..

[7] Cancer (IARC), T.I.A. for R. on Global Cancer Observatory. https://gco.iarc.fr/.

[8] Vilchis-Landeros M.M., Vázquez-Meza H., Vázquez-Carrada M., Uribe-Ramírez D., Matuz-Mares D. (2024). Antioxidant Enzymes and Their Potential Use in Breast Cancer Treatment. Int. J. Mol. Sci..

[9] De Luca F., Roda E., Ratto D., Desiderio A., Venuti M.T., Ramieri M., Bottone M.G., Savino E., Rossi P. (2023). Fighting Secondary Triple-Negative Breast Cancer in Cerebellum: A Powerful Aid from a Medicinal Mushrooms Blend. Biomed. Pharmacother..

[10] Nava-Tapia D.A., Román-Justo N.Y., Cuenca-Rojo A., Guerrero-Rivera L.G., Patrón-Guerrero A., Poblete-Cruz R.I., Zacapala-Gómez A.E., Sotelo-Leyva C., Navarro-Tito N., Mendoza-Catalán M.A. (2024). Exploring the Potential of Tocopherols: Mechanisms of Action and Perspectives in the Prevention and Treatment of Breast Cancer. Med. Oncol..

[11] Lewandowska A., Rudzki M., Rudzki S., Lewandowski T., Laskowska B. (2019). Environmental Risk Factors for Cancer—Review Paper. Ann. Agric. Environ. Med..

[12] Sadoughi F., Dana P.M., Asemi Z., Shafabakhash R., Mohammadi S., Heidar Z., Mirzamoradi M., Targhazeh N., Mirzaei H. (2022). Molecular and Cellular Mechanisms of Melatonin in Breast Cancer. Biochimie.

[13] Li S., Wu H., Chen M., Tollefsbol T.O. (2023). Paternal Combined Botanicals Contribute to the Prevention of Estrogen Receptor–Negative Mammary Cancer in Transgenic Mice. J. Nutr..

[14] Liedtke C., Mazouni C., Hess K.R., André F., Tordai A., Mejia J.A., Symmans W.F., Gonzalez-Angulo A.M., Hennessy B., Green M. (2008). Response to Neoadjuvant Therapy and Long-Term Survival in Patients With Triple-Negative Breast Cancer. J. Clin. Oncol..

[15] Lehmann B.D., Bauer J.A., Chen X., Sanders M.E., Chakravarthy A.B., Shyr Y., Pietenpol J.A. (2011). Identification of Human Triple-Negative Breast Cancer Subtypes and Preclinical Models for Selection of Targeted Therapies. J. Clin. Investig..

[16] Newton E.E., Mueller L.E., Treadwell S.M., Morris C.A., Machado H.L. (2022). Molecular Targets of Triple-Negative Breast Cancer: Where Do We Stand?. Cancers.

[17] Nussbaumer S., Bonnabry P., Veuthey J.-L., Fleury-Souverain S. (2011). Analysis of Anticancer Drugs: A Review. Talanta.

[18] Majrashi T.A., Alshehri S.A., Alsayari A., Muhsinah A.B., Alrouji M., Alshahrani A.M., Shamsi A., Atiya A. (2023). Insight into the Biological Roles and Mechanisms of Phytochemicals in Different Types of Cancer: Targeting Cancer Therapeutics. Nutrients.

[19] Hecht F., Pessoa C.F., Gentile L.B., Rosenthal D., Carvalho D.P., Fortunato R.S. (2016). The Role of Oxidative Stress on Breast Cancer Development and Therapy. Tumor Biol..

[20] Singh A.K., Yu X. (2020). Tissue-Specific Carcinogens as Soil to Seed BRCA1/2-Mutant Hereditary Cancers. Trends Cancer.

[21] Sharifi M., Moridnia A., Mortazavi D., Salehi M., Bagheri M., Sheikhi A. (2017). Kefir: A Powerful Probiotics with Anticancer Properties. Med. Oncol..

[22] De Luca F., Roda E., Rossi P., Bottone M.G. (2024). Medicinal Mushrooms in Metastatic Breast Cancer: What Is Their Therapeutic Potential as Adjuvant in Clinical Settings?. Curr. Issues Mol. Biol..

[23] Page M.J., McKenzie J.E., Bossuyt P.M., Boutron I., Hoffmann T.C., Mulrow C.D., Shamseer L., Tetzlaff J.M., Akl E.A., Brennan S.E. (2021). The PRISMA 2020 Statement: An Updated Guideline for Reporting Systematic Reviews. BMJ.

[24] Forcados G.E., James D.B., Sallau A.B., Muhammad A., Mabeta P. (2017). Oxidative Stress and Carcinogenesis: Potential of Phytochemicals in Breast Cancer Therapy. Nutr. Cancer.

[25] Herdiana Y., Sriwidodo S., Sofian F.F., Wilar G., Diantini A. (2023). Nanoparticle-Based Antioxidants in Stress Signaling and Programmed Cell Death in Breast Cancer Treatment. Molecules.

[26] Tafazoli A. (2017). Coenzyme Q10 in Breast Cancer Care. Future Oncol..

[27] Li Y., Lin Q., Lu X., Li W. (2021). Post-Diagnosis Use of Antioxidant Vitamin Supplements and Breast Cancer Prognosis: A Systematic Review and Meta-Analysis. Clin. Breast Cancer.

[28] Eren E., Das J., Tollefsbol T.O. (2024). Polyphenols as Immunomodulators and Epigenetic Modulators: An Analysis of Their Role in the Treatment and Prevention of Breast Cancer. Nutrients.

[29] Sindhu R.K., Verma R., Salgotra T., Rahman M.H., Shah M., Akter R., Murad W., Mubin S., Bibi P., Qusti S. (2021). Impacting the Remedial Potential of Nano Delivery-Based Flavonoids for Breast Cancer Treatment. Molecules.

[30] Ezzati M., Yousefi B., Velaei K., Safa A. (2020). A Review on Anti-Cancer Properties of Quercetin in Breast Cancer. Life Sci..

[31] Bartolacci C., Andreani C., Amici A., Marchini C. (2018). Walking a Tightrope: A Perspective of Resveratrol Effects on Breast Cancer. Curr. Protein Pept. Sci..

[32] Scafuri L., Buonerba C., Strianese O., De Azambuja E., Palleschi M., Riccio V., Marotta V., Scocca C., Riccio G., Errico C. (2025). Impact of Dietary Supplements on Clinical Outcomes and Quality of Life in Patients with Breast Cancer: A Systematic Review. Nutrients.

[33] Samavat H., Ursin G., Emory T.H., Lee E., Wang R., Torkelson C.J., Dostal A.M., Swenson K., Le C.T., Yang C.S. (2017). A Randomized Controlled Trial of Green Tea Extract Supplementation and Mammographic Density in Postmenopausal Women at Increased Risk of Breast Cancer. Cancer Prev. Res..

[34] Xiang L.-P., Wang A., Ye J.-H., Zheng X.-Q., Polito C., Lu J.-L., Li Q.-S., Liang Y.-R. (2016). Suppressive Effects of Tea Catechins on Breast Cancer. Nutrients.

[35] Zhao H., Zhu W., Jia L., Sun X., Chen G., Zhao X., Li X., Meng X., Kong L., Xing L. (2016). Phase I Study of Topical Epigallocatechin-3-Gallate (EGCG) in Patients with Breast Cancer Receiving Adjuvant Radiotherapy. Br. J. Radiol..

[36] Yu Z., Samavat H., Dostal A.M., Wang R., Torkelson C.J., Yang C.S., Butler L.M., Kensler T.W., Wu A.H., Kurzer M.S. (2017). Effect of Green Tea Supplements on Liver Enzyme Elevation: Results from a Randomized Intervention Study in the United States. Cancer Prev. Res..

[37] Markowska A., Antoszczak M., Markowska J., Huczyński A. (2025). Role of Epigallocatechin Gallate in Selected Malignant Neoplasms in Women. Nutrients.

[38] Diallo A., Deschasaux M., Partula V., Latino-Martel P., Srour B., Hercberg S., Galan P., Fassier P., Guéraud F., Pierre F.H. (2016). Dietary Iron Intake and Breast Cancer Risk: Modulation by an Antioxidant Supplementation. Oncotarget.

[39] Skouroliakou M., Grosomanidis D., Massara P., Kostara C., Papandreou P., Ntountaniotis D., Xepapadakis G. (2018). Serum Antioxidant Capacity, Biochemical Profile and Body Composition of Breast Cancer Survivors in a Randomized Mediterranean Dietary Intervention Study. Eur. J. Nutr..

[40] Zick S.M., Colacino J., Cornellier M., Khabir T., Surnow K., Djuric Z. (2017). Fatigue Reduction Diet in Breast Cancer Survivors: A Pilot Randomized Clinical Trial. Breast Cancer Res. Treat..

[41] Hosseinzadeh A., Alinaghian N., Sheibani M., Seirafianpour F., Naeini A.J., Mehrzadi S. (2024). Melatonin: Current Evidence on Protective and Therapeutic Roles in Gynecological Diseases. Life Sci..

[42] Stephenson E., Mclaughlin M., Bray J.W., Saxton J.M., Vince R.V. (2024). Nutrition Modulation of Cardiotoxicity in Breast Cancer: A Scoping Review. Nutrients.

[43] Mohseni H., Amani R., Hosseini S.A., Ekrami A., Ahmadzadeh A., Latifi S.M. (2019). Genetic Variations in VDR Could Modulate the Efficacy of Vitamin D3 Supplementation on Inflammatory Markers and Total Antioxidant Capacity among Breast Cancer Women: A Randomized Double Blind Controlled Trial. Asian Pac. J. Cancer Prev..

[44] Fatemi Shandiz A., Karimi G., Dayyani M., Hosseini S., Elyasi S. (2025). Evaluation of Oral Silymarin Formulation Efficacy in Prevention of Doxorubicin Induced Hepatotoxicity in Patients with Non-Metastatic Breast Cancer. J. Oncol. Pharm. Pract..

[45] Farnoosh G., Saeedi-Boroujeni A., Jalali A., Keikhaei B., Mahmoudian-Sani M.-R. (2021). Polymorphisms in Genes Involved in Breast Cancer Among Iranian Patients. Pers. Med..

[46] Summer M., Sajjad A., Ali S., Hussain T. (2024). Exploring the Underlying Correlation between Microbiota, Immune System, Hormones, and Inflammation with Breast Cancer and the Role of Probiotics, Prebiotics and Postbiotics. Arch. Microbiol..

[47] German R., Marino N., Hemmerich C., Podicheti R., Rusch D.B., Stiemsma L.T., Gao H., Xuei X., Rockey P., Storniolo A.M. (2023). Exploring Breast Tissue Microbial Composition and the Association with Breast Cancer Risk Factors. Breast Cancer Res..

[48] Thu M.S., Ondee T., Nopsopon T., Farzana I.A.K., Fothergill J.L., Hirankarn N., Campbell B.J., Pongpirul K. (2025). Correction: Thu et al. Effect of Probiotics in Breast Cancer: A Systematic Review and Meta-Analysis. *Biology*
**2023**, *12*, 280. Biology.

[49] Ambrosone C.B., Zirpoli G.R., Hutson A.D., McCann W.E., McCann S.E., Barlow W.E., Kelly K.M., Cannioto R., Sucheston-Campbell L.E., Hershman D.L. (2020). Dietary Supplement Use During Chemotherapy and Survival Outcomes of Patients with Breast Cancer Enrolled in a Cooperative Group Clinical Trial (SWOG S0221). J. Clin. Oncol..

[50] Reitz L.K., Baptista S.D.L., Santos E.D.S., Hinnig P.F., Rockenbach G., Vieira F.G.K., De Assis M.A.A., Da Silva E.L., Boaventura B.C.B., Pietro P.F.D. (2020). Diet Quality Is Associated with Serum Antioxidant Capacity in Women with Breast Cancer: A Cross Sectional Study. Nutrients.

[51] Guo C.-H., Hsia S., Chung C.-H., Lin Y.-C., Shih M.-Y., Chen P.-C., Peng C.-L., Henning S.M., Hsu G.-S.W., Li Z. (2021). Nutritional Supplements in Combination with Chemotherapy or Targeted Therapy Reduces Tumor Progression in Mice Bearing Triple-Negative Breast Cancer. J. Nutr. Biochem..

[52] Greenlee H., Kwan M.L., Kushi L.H., Song J., Castillo A., Weltzien E., Quesenberry C.P., Caan B.J. (2012). Antioxidant Supplement Use after Breast Cancer Diagnosis and Mortality in the Life After Cancer Epidemiology (LACE) Cohort. Cancer.

[53] Hu W., Lyu X., Xu H., Guo X., Zhu H., Pan H., Wang L., Yang H., Gong F. (2023). Intragastric Safflower Yellow Alleviates HFD Induced Metabolic Dysfunction-Associated Fatty Liver Disease in Mice through Regulating Gut Microbiota and Liver Endoplasmic Reticulum Stress. Nutrients.

[54] Kumar N.B. (2021). The Promise of Nutrient-Derived Bioactive Compounds and Dietary Components to Ameliorate Symptoms of Chemotherapy-Related Cognitive Impairment in Breast Cancer Survivors. Curr. Treat. Options Oncol..

[55] Shi D.-D., Dong C.M., Ho L.C., Lam C.T.W., Zhou X.-D., Wu E.X., Zhou Z.-J., Wang X.-M., Zhang Z.-J. (2018). Resveratrol, a Natural Polyphenol, Prevents Chemotherapy-Induced Cognitive Impairment: Involvement of Cytokine Modulation and Neuroprotection. Neurobiol. Dis..

[56] Al-Hammadi N., AlSabri E., Kudhair A.H., Qassam H., Hadi N.R. (2023). Coq10 for Preventing Cardiotoxicity in Breast Cancer Patients Treated with Trastuzumab. J. Med. Life.

[57] Otles S., Cagindi O. (2003). Kefir: A Probiotic Dairy-Composition, Nutritional and Therapeutic Aspects. Pak. J. Nutr..

[58] Liu H., Lee J.I., Ahn T.-G. (2019). Effect of Quercetin on the Anti-Tumor Activity of Cisplatin in EMT6 Breast Tumor-Bearing Mice. Obstet. Gynecol. Sci..

[59] Mitchell M.J., Billingsley M.M., Haley R.M., Wechsler M.E., Peppas N.A., Langer R. (2021). Engineering Precision Nanoparticles for Drug Delivery. Nat. Rev. Drug Discov..

[60] Yang B., Dong Y., Wang F., Zhang Y. (2020). Nanoformulations to Enhance the Bioavailability and Physiological Functions of Polyphenols. Molecules.

[61] Khalil I., Yehye W.A., Etxeberria A.E., Alhadi A.A., Dezfooli S.M., Julkapli N.B.M., Basirun W.J., Seyfoddin A. (2019). Nanoantioxidants: Recent Trends in Antioxidant Delivery Applications. Antioxidants.

[62] Priya S., Desai V.M., Singhvi G. (2023). Surface Modification of Lipid-Based Nanocarriers: A Potential Approach to Enhance Targeted Drug Delivery. ACS Omega.

[63] Bae J.-M. (2016). Reinterpretation of the Results of a Pooled Analysis of Dietary Carotenoid Intake and Breast Cancer Risk by Using the Interval Collapsing Method. Epidemiol. Health.

